# Passant Connection Screw of Dental Implants: An *In Vitro* SEM Preliminary Study

**DOI:** 10.1155/2022/9720488

**Published:** 2022-04-21

**Authors:** Gabriele Cervino, Antonino Germanà, Luca Fiorillo, Cesare D'Amico, Francesco Abbate, Marco Cicciù

**Affiliations:** ^1^School of Dentistry Department of Biomedical and Dental Sciences and Morphofunctional Imaging, University of Messina, Via Consolare Valeria, 1, 98125 Messina, Italy; ^2^Department of Veterinary Sciences, University of Messina, 98168 Messina, Italy

## Abstract

The use of dental implants in oral rehabilitations has become increasingly common, thanks to the safety and predictability of these rehabilitations. Unfortunately, dental implants, being alloplastic devices, are not free from biomechanical complications, especially in the case in which the connections are complex and involve several components. The aim of the study is to highlight what could be surface alterations using different screwing torques, or by repeating the screwing process several times. In this study, 40 passant screws (Osstem®, South Korea Dental Implant Ebony Gold®) were examined under a Zeiss EVO LS 10 scanning electron microscope (SEM), operating with an accelerating voltage of 20 kV. Passant screws were subdivided into 4 groups: 30 Nmm tightening torque; maximum tightening torque; 2 times 30 Nmm tightening torque; no screwing, new ones (control group). There are no significant differences in the surfaces of the passant screws in SEM images, and the 100% of the passant screws is free of defects or fractures. Surely, further studies and investigations will certainly be needed to allow improvement of these devices.

## 1. Introduction

The use of dental implants has several advantages in dentistry. Additionally, the efficiency and clinical success of osseointegration have been reported by numerous articles, reporting that dental implants have become a common treatment for tooth/teeth loss [1, 2]. Going back in time, problems associated with tooth loss since in ancient times could be mentioned. On the contrary, in modern times, aesthetic factors have acquired importance in the maintenance of one's teeth and with the evolution of contemporary dental techniques, the replacement of missing teeth has become possible and enforced. Over hundreds of centuries, methods of managing tooth loss have evolved, in which initial attempts were made to transplant teeth from one individual to another; and different strategies for designing an anchored tooth root shape or other substructure have been tested [3]. Advances in technology allowed efficient assistance with the positioning of the implant, with high accuracy and precision for each patient. Nevertheless, the need for a thorough physical examination of patients to provide them with a solution to their problems has been constant over time [4]. During the 1980s and 1990s, threadless cylindrical implants were designed and incorporated, based on plasma sprayed titanium surface coatings or hydroxyapatite ones. On the other hand, in a group of patients has been reported a crestal bone loss resulting in implant failure [5, 6]. While, currently, most implants inserted have a threaded design rather than a cylindrical “press-fit” design [7].

Meanwhile, over the last decade, an evolution of dental implants has been observed due to the development and research for improving of patient care [8]. As a success of the implant, osseointegration has been always considered a fundamental and priority factor [9].

Interestingly, there have been indicated different complications after several years of follow-up on a single implant prosthesis. Despite the high clinical success of the implant, various complications should be considered, such as fracture of the abutment screw, inflammation of the mucosa, and penetration of soft tissues, and the most common complication was abutment screw loosening [10–12]. Shortly, it could be emphasized that the cause of a screw loosening is, first of all, fatigue, an inadequate tightening torque, a settling effect, a microvibratory movement, and/or an excessive flexion [13]. Other studies have stated that the application of a tightening torque to the abutment screw was aimed at obtaining an elongation of the screw and ensuring stability through a compressive force between the abutment system and the fixture [14]. It is important to underline that the dental implant abutments must be made of biocompatible materials with adequate mechanical properties to meet biological, functional, and aesthetic needs [15]. In particular, they must fit accurately and passively on their implants to prevent the unwanted complications, such as screw loosening, abutment fractures, and bone loss during implant surgery. For optimal mucogingival esthetics, dental implant abutments also require the appropriate emergence profile needed to support the surrounding soft tissue [16].

As well known, removable and fixed prostheses have been—and still are—used to restore chewing function and aesthetics as well. For this, dental implants have become an additional treatment option to replace missing tooth/teeth and the respective treatment concepts have reported high success rates of 99.7% and 89.5% in 10 [17] and 20 years [18], respectively. As in any medical intervention, biological complications could occur, which could ultimately lead in this case to complete implant failure and consequently, in the worst case, the implant's removal. Implant failure, in general, can be described as an early or late event depending on the characterization of the time point [18].

Surely, these success rates, especially in recent years, tend to be higher. This is because there has been an evolution in the materials and biomechanics of dental implants. It is enough to consider, for example, the large differences between dental implants, which provided for cemented abutments, and those which provided for screwed. Already in this there are big differences from a clinic and survival point of view. Also, with regard to the passing screws themselves, the subject of this study, it is said that rescrewing of a loosened abutment should not be done because it can increase the risk of a fracture of the abutment [12–18].

In addition, early failures can have multiple causes, i.e., overheating of the bone during implant site preparation, lack of primary stability due to excessive site preparation or poor bone quality, overload, or parafunctions [16], reported in this way, before the implants are functionally loaded and then mainly represent inadequate healing and osseointegration in the initial phase. Consequently, the implants are clinically mobile and easy to be removed. Therefore, late failures after loading and function may be reported for biological reasons, such as bone loss due to peri-implantitis or implant fractures. Sometimes, even a healthy and osseointegrated implant is considered a failure due to extreme malpositioning and prosthetic reasons. It is better, that in this case, the removal of the implant is recommended. Not so pleasant, is the case in which a late failure of the nonmobile and partially osseointegrated implant is highlighted in the apical region. It is important to underline that the attempt to remove this type of implant can be very challenging, and the removal must necessarily be less invasive as possible to prevent the teeth and adjacent structures from being damaged [17, 18].

## 2. Materials and Methods

### 2.1. Geometries of Dental Implants, Abutments, and Connection Screws

With the evolution of implant types, different designs of the implant-abutment connection have been introduced [19]. According to the literature [20, 21], the first osseointegrated implants had an external hexagonal design on their platform. A lot of cases with bone loss, due to chronic inflammation of the implant-abutment interface, have been reported, hypothesizing on the distribution of tensions in the marginal bone crest and on the presence of micromovements at the implant-abutment interface [22, 23].

To exceed some design limitations and bone loss of the external hex connection, internal connections were introduced, with the aim to provide better esthetics, joint strength, microbial sealing, and long-term stability [22–24]. One type of internal connection is the Morse taper one, which creates friction between surfaces through cold welding [25]. So, this connection provides a greater surface area of the implant-abutment interface, producing a good seal between its components and fewer microinfiltrations, giving superior joint stability and less marginal bone loss [26].

Notwithstanding by the geometric configuration type, the prosthetic abutment will be fixed to the implant through a screw, leading an interface between the implant-abutment junction, while the inevitable gap that can be created between implant and abutment can cause biological complications by the bacteria infiltration and/or their metabolic products towards the connection [27]. In addition, this presence of the gap can incorrectly transmit the forces from the abutment to the implant, provoking constant micromovements, which over the time can cause biomechanical complications, such as loose abutment screw, screw or abutment rotation, and/or fracture and a reduction of the prosthetic screw preload [28].

Mostly, the filtration degree between the implant and its prosthetic components depends on several factors, such as the geometry connection, an accurate fit between the components, the freedom of abutment rotation on the implant, the applied torque load to tighten the abutment, and the micromovements between the components of the implant-abutment complex [24, 26, 29]. In this study, 40 passant screws were examined (Osstem®, South Korea Dental Implant Ebony Gold®). These screws are subjected to the “E.L.I. Electric Low Interstitial” treatment. This is a treatment that eliminates the surface irregularities due to the turning process, thanks to which, it increases the resistance of the screw to yield and therefore to fracture. Screw thread evaluation was performed under SEM after washing (all retrieved specimens were washed) for 10 minutes using ultrasonic cleaning equipment.

**Figure 1:**
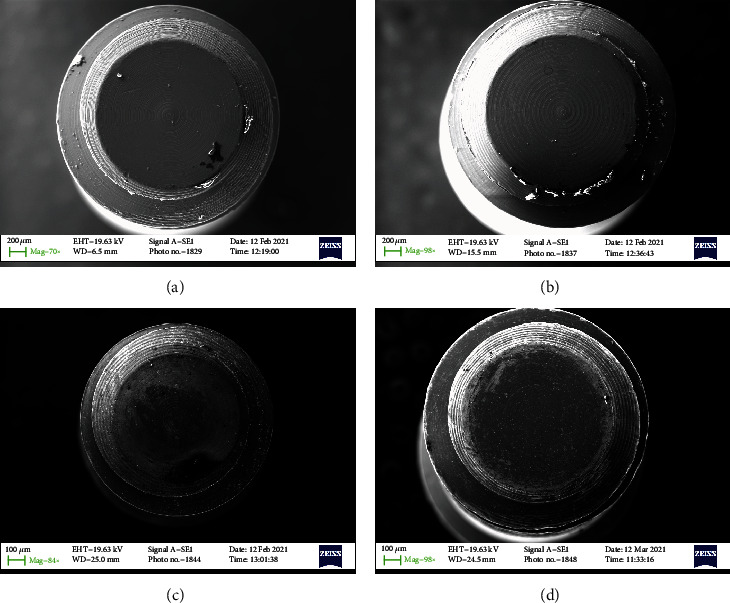
SEM detail of the head of the connection screw. (a) A force of 30 Nmm (screwed); (b) Max torque (screwed); (c) Two times 30 N (screwed); (d) Control screw (not screwed).

**Figure 2:**
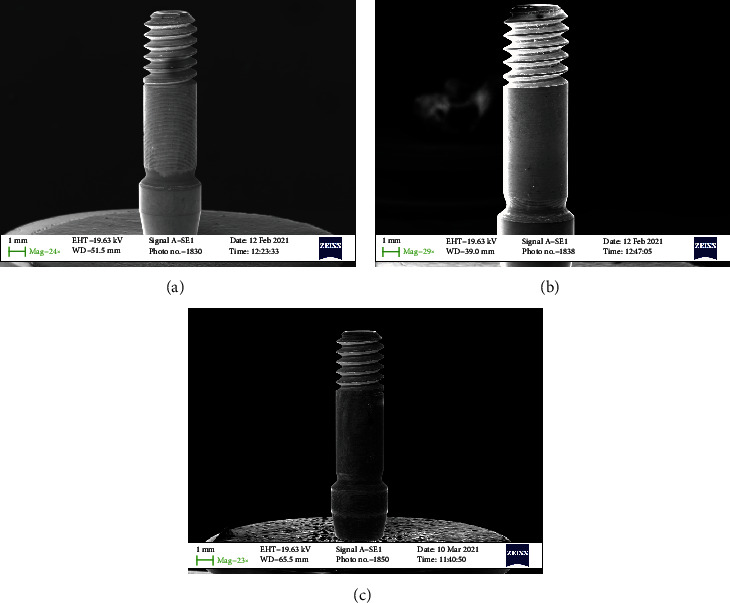
SEM detail of the connection screw. (a) A force of 30 Nmm (screwed); (b) Max torque (screwed); (c) Control screw (not screwed).

**Figure 3:**
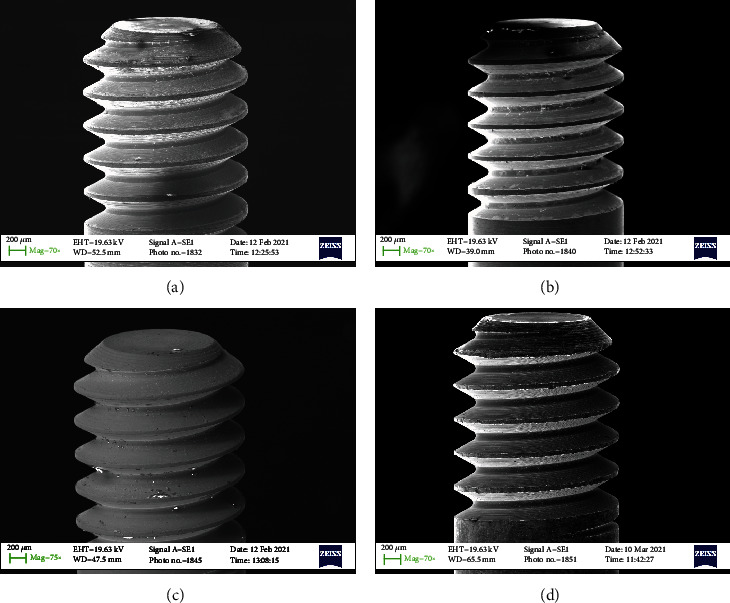
SEM detail of the head of the connection screw. (a) A force of 30 N (screwed); (b) Max torque (screwed); (c) Two times 30 N (screwed); (d) Control screw (not screwed).

**Figure 4:**
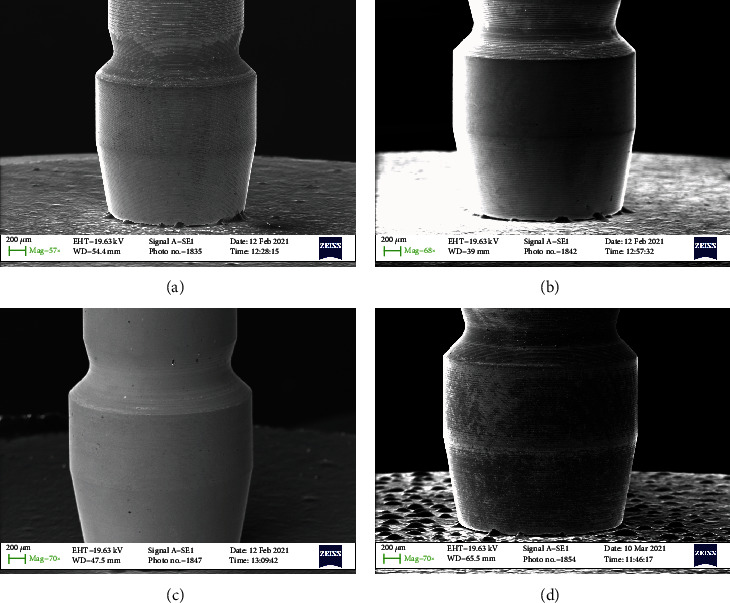
SEM detail of the top of the connection screw. (a) A force of 30 N (screwed); (b) Max torque (screwed); (c) Two times 30 N (screwed); (d) Control screw (not screwed).

All samples were subdivided into 4 groups of 10 screws each (Figures 1–4(a)–(d)).

- Group 1 with 30 Nmm tightening torque
- Group 2 with max tightening torque
- Group 3 with 2 times 30 Nmm tightening torque
- Group 4 the control group

Tightening force was assessed manually with the use of an Osstem®, South Korea Dental Implant dynamometric key.

### 2.2. SEM Study

The samples were examined under a Zeiss EVO LS 10 scanning electron microscope, operating with an accelerating voltage of 20 kV, after first visualization and analysis with an optical microscope, with the aim of highlighting large surface defects. The same samples were evaluated under scanning electron microscopy, in order to observe and/or evaluate the metal microstructure and the cracks. This type of scanning electron microscope does not need any metallization of the surface.

## 3. Results

### 3.1. Tightening

Tightening the through screws of the abutments, MUAs, and turrets allows a perfect coupling between abutment and fixture. Ensuring to minimize the micromovements of the abutment or MUA on the implant.

These incongruous movements can lead to repeated implant trauma and therefore the onset of peri-implantitis, or even in the early phase of immediate loading, the loss of osseointegration and therefore the loss of the implant.

Therefore, it is important during the prosthetic phase, in deferred loading or in the immediate loading phase, to tighten the through screws with a torque between 20/35 Newton. Consequently, it is essential to know the diameter of the through screw, the thread, and the length of the screw to ensure optimal tightening.

To avoid errors, it is important to use a dynamometric key. Skipping these steps could lead to early or late implant failure.

### 3.2. The Connecting Screw

The union of the abutments and/or the prosthesis on the dental implant takes place via the connection or passant screw. In the case of the screwed prosthesis, the connection screw is used either for direct fixing of the prosthesis on the implant or for fixing the abutment on the implant and then (via a second screw) for fixing the prosthesis on the abutment. On the other hand, in the cemented prosthesis, the connection screw is used only to fix the abutment on the implant and then the prosthesis is cemented on the abutment.

In any case, the problem that often arises in the use of the connection screw is to guarantee the reliability of the tightening, preventing it from breaking in use or unscrewing over time. The tightness of the screw depends on many mechanical aspects. Firstly, the screw is initially inserted into its housing and then screwing is initiated during which the rotation motion of the screw is transformed into a feed motion, which allows the screw to enter the threaded hole. This phase takes place without significant friction and therefore applying a very low torque. As for Figures 1–4 no macro- or microfracture of the passant screw can be observed in the groups, so is possible to state a 100% of success rate of these passant screw with different protocols.

## 4. Discussion

### 4.1. Dental Implant Failures

Substantially, the accuracy of fit at the implant-abutment interface is an important key for long-term osseointegration and the success of dental implants. Mainly, maladjustment at the implant-abutment interface can produce biological (microbial colonization, bone loss, and loss of implant osseointegration) and mechanical complications (screw loose/fracture and abutment fracture) [1, 3, 7, 12, 13, 15]. Several authors have pointed out that the presence of microgaps in the implant-abutment connection depends on various factors, such as the imprecise mechanization of the implant connection, inadequate implant-abutment adaptation, or the applied torque load, leading in this way the implant failure [30–39].

### 4.2. Fractures of Dental Implants

In the literature, dental implant has been described in a lot the implant fracture, including, here, possible causes, such as bruxism, large occlusal forces, mechanical trauma, small implant diameter, material fatigue, and advanced bone loss, leading to reduced mechanical support of the implant. Other authors have described the risk of implant fractures with a low prevalence of 1%, thus considering a risk especially in the molar region [40].

Recently, dental implant was published on implant fractures, evaluating 19,087 implants in 8,501 patients and observing fractures in 70 implants (0.4%), whereas, for zirconia implants, less data is available, and for the latter, some authors evaluated the fracture rate of zirconia implants in a systematic review [41]. Principally, the focus is directed on implants available on the market, where the fracture rate has again dropped to 0.2%.

In the daily clinic, different methods of implant removal are encountered, including, here, the use of the implant ratchet [12], piezoelectric surgery [42], high-speed drills [40], elevators, forceps, trephine burs, and laser surgery.

### 4.3. Fractures of the Connecting Screws

Frequently, connecting screw is discussed in the fracture of prosthetic connecting screw that years ago was considered a fairly common complication in implantology [4, 12]. While, according to some other authors, the most common reasons highlighted were inadequate or excessive preload, loosening of screws, and unfavorable forces on implant components [6, 8]. When the head of the screw reaches the stop, it cannot move forward and it becomes difficult to screw it further. If a high torque is applied, the screw rotates again, and part of the screw descends inside the threaded hole, but the head remains in contact with the abutment, without following the thread. This produces an elongation of the shaft of the screw (the shaft is that part between the head and the first thread in the grip). This elongated shaft is subjected to a state of stress that is greater than the small resistant section (diameter of the screw). However, the increase in the tightening torque increases the pulling force and the strain in the shaft. It is important that the stress remains in the elastic range of the material from which the screw is made. If the torque is excessive, the effort exceeds the elastic limit, and therefore, a plastic deformation of the shaft of the screw is produced, but, above all, the screw is in contact with the threads. In this way, there is a reduction in the contact force and damage to the screw itself: it is no longer able to maintain the correct tightening. Both insufficient and excessive tightening can therefore lead to unscrewing of the screw. In the first case, the screw is progressively unscrewed because the friction opposing the rotation is insufficient with respect to the torsional torques randomly deriving from the chewing loads. Then, in the second case, the screw is unscrewed because its plasticization reduces its sealing capacity, as previously described.

In general, however, the ideal load is the one that allows to obtain the average effort and this depends on the material, the section of the screw, and the initial length of the screw.

Gear errors must also be considered. These usually are in relation to the manufacturing that can be classified into

- pitch errors
- eccentricity
- errors in the profile (normal or modified)

A single pitch error consists of incorrect spacing between two consecutive teeth. For high-performance gears produced with advanced technologies, the maximum pitch error between adjacent teeth can be quantified in the order of a deviation of few microns of the pitch from the nominal value.

The eccentricity of a gear determines a behavior similar to that of a series of pitch errors. Eccentricity for high performance gears should not exceed 20 *μ*m. In practice, the effect of eccentricity is to generate, in the absence of “real” pitch errors, an “apparent” pitch error which varies cyclically at each rotation. The magnitude of the maximum “apparent” pitch error can be easily evaluated geometrically and is equal to 2ecsin (*π*/Z).

In the errors in the geometry of the profile, we can say that with the current technologies for high performance gears, there are deviations from the “drawing” geometry of the order of the measurable quantities of the verification tools; therefore, in a study that neglects the effects of profile errors, it can be considered exhaustive (at least in the context of high-level design). How to solve and overcome the inaccuracy between thread and threaded pitch in order to reach a window that engages the whole screw and is not expressed only in a “traction” between the head and the first turn of the thread passing through the shaft of the screw itself?

A first hypothesis is to increase the thickness of the thread, making fewer turns and, therefore, a larger pitch. This would increase the tightness of the screw as in reality it is only the first thread to enter into traction and guarantee the seal between the abutment and fixture and/or abutment and prosthesis (crown). Having a long screw with many turns, if “precision” is not guaranteed, does not actually favor sealing.

A second hypothesis is to increase the precision between the thread of the screw and the threaded cable of the implant or abutment, by inserting “thread-locking” pastes or other gasket materials inside the threaded cable or on the screw thread before screwing. These could also play an important occluding function.

In the third hypothesis, the tightening torque varies according to the engagement of the screw, increasing from 15 N to 35 N. Furthermore, the screw, once tightened to a maximum of 35 N, should be replaced at each check-unscrewing, because it loses its original capacity.

In practice, they should be disposable so always change at each unscrewing or disassembly in the various controls. Furthermore, the screw should be tightened to the torque imposed by the manufacturers, on average between 65 and 75% of the yield strength of the screw, which must be reached in tightening, but never exceeded.

It is also important to wait 10 minutes after the first tightening and repeat the locking operation. This is at least three times.

Thus, Kreissl et al. found that the incidence of screw fracture over a 5-year period was up to 3.9%.

Additionally, a screw fractures before any irreversible damage to the implant or prosthesis, serving as a safety system. Anyway, several studies have described techniques to save or remove fractured screws. About this issue, various dental implant companies have their own screw recovery kits, but most of these systems use aggressive motorized devices that increase the risk of implant damage, achieving a nonrestorable implant. The first step is the removal of the coronal portion of the fractured screw, gaining a visual access to see the remaining screw fragment. In cases when the fracture is sufficiently coronal, an explorer and a hemostat can be used; otherwise, an ultrasonic scaler with a blunt tip, without damaging the internal threads of the implant. It is important to use a flathead screwdriver to insert the created groove and attempt to rotate the screw fragment counterclockwise. Unfortunately, in cases when the internal threads of the implant are damaged during the removal of the fractured screw, the implant will become useless and not restorable, with the fate to be removed.

Knowing in depth the behavior of these materials will certainly help to optimize their performance and improve their duration and therefore the predictability over time of rehabilitations in the biomedical field. This study aims to be preliminary for further analysis of this kind. Precisely for this reason, a fracturographic analysis was not carried out, which will be the result of subsequent studies. It could be interesting to test these screws under dynamic masticatory loads.

## 5. Conclusion

This study brings to light a series of information regarding implant components, especially prosthetic ones. Once, it is possible to clarify what the protocols are as follows: they are clear protocols on how an implant failure or failures linked to prosthetic components that could be derived from materials. This observational study did not show any defect or fracture through SEM analysis in different groups, stating a 100% success rate. Surely, thanks to this study and to others who follow. It will be possible to optimize these materials, so as to make them more performing and guarantee greater safety and predictability in implant-prosthetic oral rehabilitation.

## Data Availability

Data is available upon request to the corresponding authors.
